# Triple Valve Replacement in a Patient with Severe Combined Aortic and Mitral Stenosis

**DOI:** 10.7759/cureus.7412

**Published:** 2020-03-25

**Authors:** Talha Ahmed, Melsjan Shkullaku, Diljon Chahal, Murtaza Dawood, Anuj Gupta

**Affiliations:** 1 Internal Medicine, University of Maryland Medical Center, Baltimore, USA; 2 Cardiology, University of Maryland, Baltimore, USA; 3 Cardiothoracic Surgery, University of Maryland, Baltimore, USA

**Keywords:** transcatheter mitral valve-in-valve replacement, valve replacement, valve replacement surgery, aortic stenosis, mitral stenosis

## Abstract

With recent advancements and evidence in favor of transcatheter approach for valve replacements including valve-in-valve procedures, it has become a favorable choice particularly in critically ill patients. Additionally, transcatheter mitral valve-in-valve replacement (TMViVR) is emerging as a less invasive substitute for patients with early dysfunctional bioprosthetic valve. We describe the clinical course of a 52-year-old male whose initial presentation to the hospital for dyspnea on exertion secondary to combined severe aortic and mitral stenosis got complicated requiring three valvular replacement procedures with favorable outcomes.

## Introduction

In patients with a high burden of comorbidities requiring multiple valvular replacement procedures during one hospitalization, redo surgery is less desirable for a dysfunctional bioprosthetic valve [1]. Our case entails the clinical course of a patient with severe calcific aortic and mitral stenosis who required three valvular replacement procedures including transcatheter mitral valve-in-valve replacement (TMViVR). The hospital course is described and rationale behind valve replacement procedures is reviewed in the light of current evidence. We demonstrate through our case that failing surgical mitral valve (MV) in critically ill patients with multiple valvular replacements makes redo surgery almost contraindicated. A favorable mitral valvular apparatus allows for TMViVR procedure. This translates into improved clinical outcomes and hemodynamic parameters for the replaced valves with less periprocedural complication [2]. 

## Case presentation

**Figure 1:**
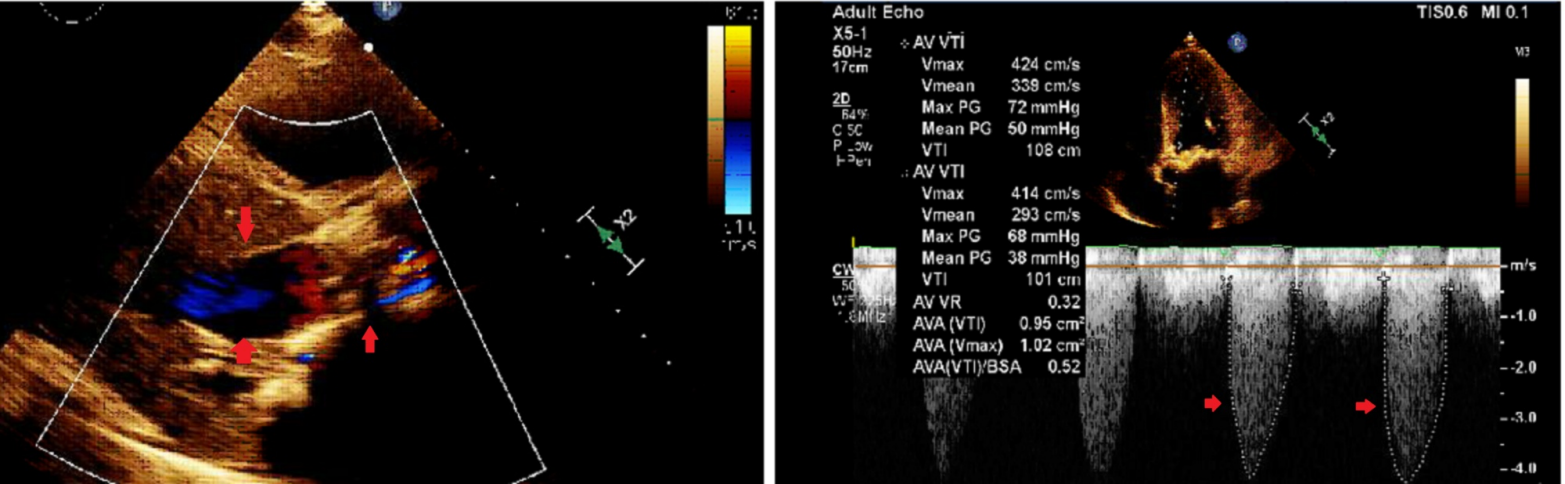
Transthoracic echocardiogram with severe aortic stenosis and elevated gradients across the valve

**Figure 2:**
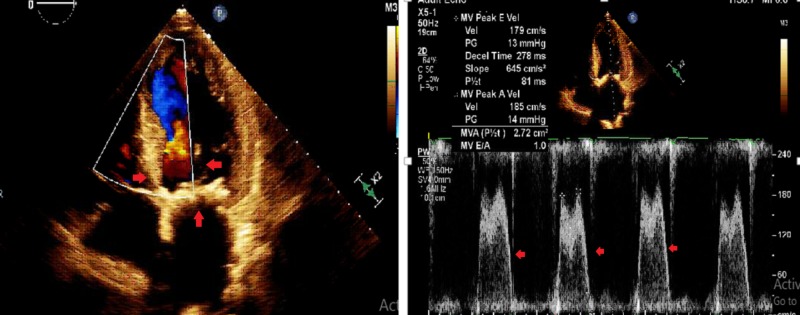
Transthoracic echocardiogram with severe mitral stenosis and elevated gradients across the valve

A 52-year-old male with a history of end-stage renal disease and hypertension presented to an outside hospital with dyspnea on exertion. Physical exam revealed holosystolic murmur at aortic and diastolic murmur at mitral area corresponding to aortic and mitral stenosis, respectively. Vital signs at presentation were stable. Electrocardiogram (EKG) and cardiac troponin were normal. A transthoracic echocardiogram (TTE) revealed severe mitral stenosis with mean and peak gradients of 11 and 29 mmHg, respectively, and severe aortic stenosis with mean and peak gradients 53 and 80 mmHg, respectively, and a valve area of 0.8 cm^2^ with left ventricle ejection fraction of 45% (Figures 1, 2). Both valves had heavily thickened and calcified leaflets.

**Figure 3:**
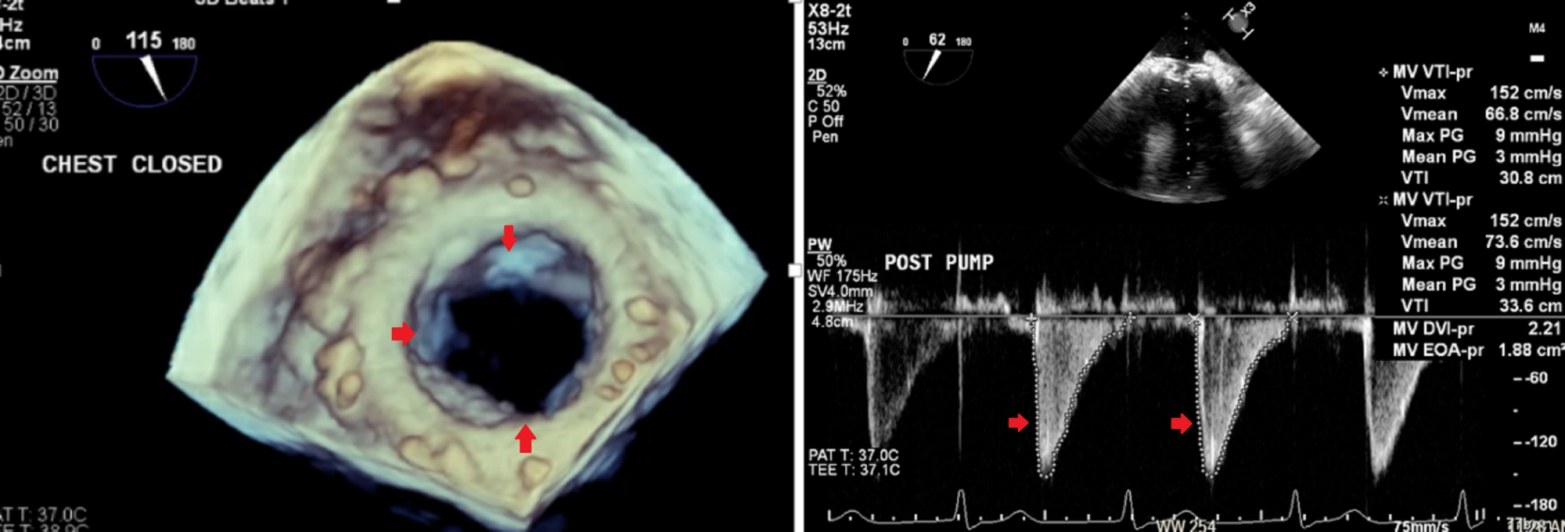
Transesophageal echocardiogram (three-dimensional view) after surgical mitral valve replacement showing normally function valve with normal gradients

Left heart catheterization was normal. The patient was referred to our facility for treatment of symptomatic valvular disease. On the day of presentation, it was decided to proceed with surgical mitral valve replacement (MVR) with a 25-mm Edwards bioprosthetic valve (Edwards Lifesciences, Irvine, CA) with plans to intervene on aortic valve as the next step (Figure 3).

**Figure 4:**
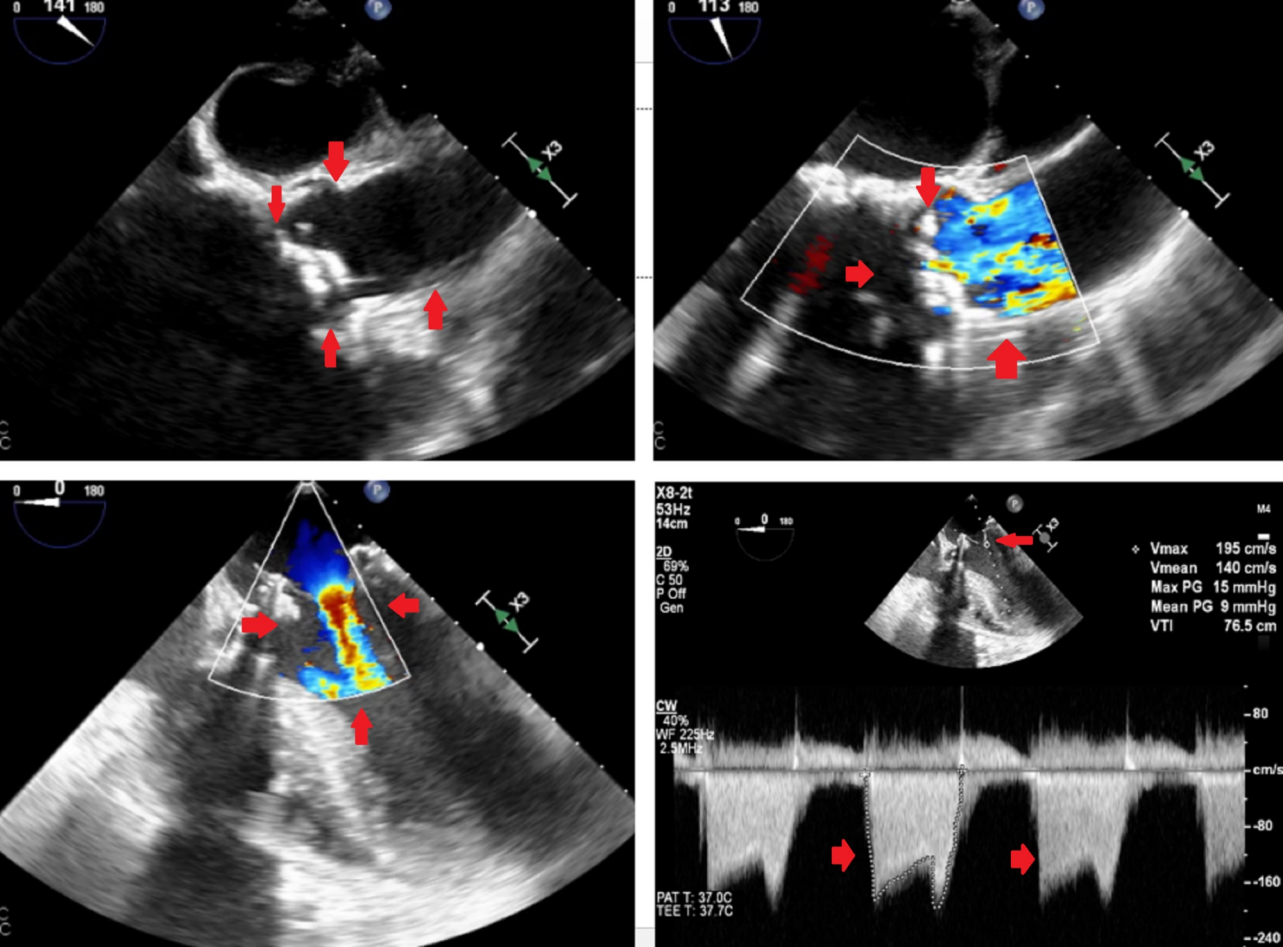
Transesophageal echocardiogram nine days after surgical mitral valve replacement with top left and right showing severe aortic stenosis and elevated gradients across the aortic valve and bottom left and right with elevated gradients across bioprosthetic mitral valve with signs of early dysfunction

The densely calcified leaflets of the aortic valve made transcatheter aortic valve replacement (TAVR) a less favorable initial procedure. On the subsequent day, however, the patient suffered a cardiac arrest (pulseless electrical activity or PEA), which was resuscitated but complicated by refractory cardiogenic shock requiring cannulation for venoarterial extracorporeal membrane oxygenation support (VA-ECMO). Due to inability to wean off ECMO combined with severely calcified aorta and high surgical risk (the Society of Thoracic Surgery/STS risk score of 12%), TAVR was eventually considered. A preoperative transesophageal echocardiogram (TEE) revealed elevated gradients across the MV bioprosthesis suggesting early dysfunction as well as confirming the severe aortic stenosis (Figure 4).

**Figure 5:**
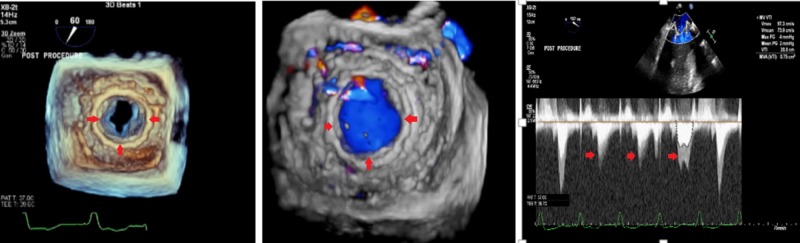
Post-transcatheter mitral valve-in-valve replacement transesophageal echocardiogram with normal gradients across the mitral valve-in-valve that opens normally in diastole

**Figure 6:**
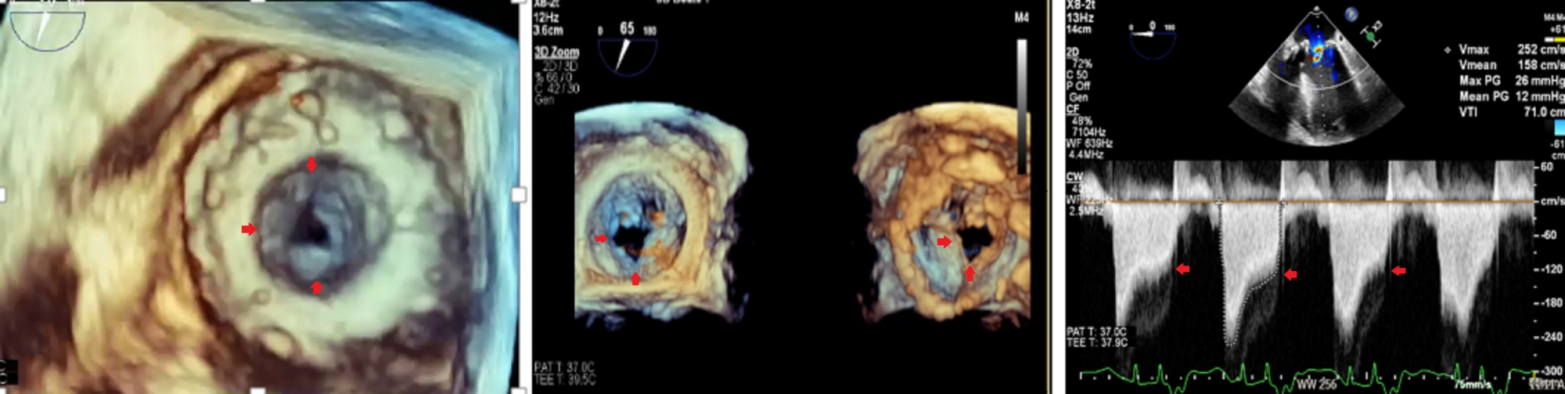
Post-transcatheter mitral valve-in-valve replacement transesophageal echocardiogram normal gradients across the aortic valve and normal valvular excursion in systole

On day 9 of presentation, TAVR was performed using the transfemoral approach to deploy a 26-mm Edwards SAPIEN 3 valve. Postoperative weaning attempts from ECMO were associated with prompt hypotension and dramatic increase in his transmitral valve gradient on subsequent TTE. Due to unclear etiology of this bioprosthesis dysfunction, high-dose anticoagulant was tried considering possibility of leaflet thrombosis associated stasis but with no improvement. The patient was ultimately referred for TMViVR due to failure in improvement of clinical status and evidence of dysfunctional bioporsthetic MV coupled with high surgical risk (STS risk score of 30%). Using a transcatheter-transseptal approach, a 26-mm SAPIEN-3 valve was placed in valve-in-valve position. Intraoperative TEE showed normally functioning mitral valve-in-valve and replaced aortic valve (Figures 5, 6).

The patient was successfully decannulated on the operating table and later discharged to a postacute facility. A follow-up echocardiogram two months later showed normally functioning mitral valve-in-valve and mild valvular and paravalvular aortic regurgitation. 

## Discussion

The recent paradigm shift to transcatheter valve replacement from surgical valve replacement provides an opportunity to perform multiple procedures gauged towards improving clinical status. The treatment of failing surgical mitral bioprostheses, however, remains a challenge. Early failure defined by elevated gradients across the valve with decrease valvular excursion within days of implantation poses its own difficulties. While recovering from the first surgery, redo surgery poses a high risk and is much less desired [3]. Our patient who had an initial surgical MVR complicated by refractory cardiogenic shock unfortunately required VA-ECMO. With a hope to wean off the VA-ECMO, TAVR was performed considering prohibitive surgical risks. However, post-TAVR course did not improve as worsening transmitral gradients were seen across the MV with decreased valvular excursion. After two different valvular procedures, failure of improvement in clinical status and evidence of early failure of surgical MVR necessitated a decision for TMViVR under the VA-ECMO support. There is a growing body of literature that supports the use of TMViVR in patients with failed surgical valve and with acceptable surgical risks. This primarily is guided by results of recent studies showing favorable outcomes when compared to redo surgery especially in high-risk patients [4,5]. The relatively low rate of periprocedural morbidity and mortality and favorable echocardiographic parameters of valve performance support the use of TMViVR as the preferred therapy in many patients with bioprosthetic mitral valve failure [6-8]. Hu et al. in their meta-analysis of patients from case reports and original articles on TMViVR concluded excellent short‐term clinical outcomes in patients with mitral stenosis, but long-term follow‐up data are currently lacking to determine the durability of the procedure [8]. In a very recent analysis of one-year outcomes of TMViVR, the authors concluded that using the SAPIEN 3 valve for the procedure is associated with high technical success, low complication rate, and significant improvement of symptoms and quality of life, which were maintained at one year. They also showed that valve performance was maintained at one year. Transseptal access was associated with lower mortality compared with transapical access and was an independent predictor of lower mortality at one year [9]. There have been reports in the past on double valve replacement including double valve-in-valve replacement procedures for combined mitral and aortic bioprosthetic valve dysfunction [10]. However, our case was unique and challenging as the patient was relatively young and underwent three valvular procedures during the same hospitalization with an improved clinical outcome.

## Conclusions

Our case reflects that with rapidly growing use of percutaneous valve replacement strategies, multiple valvular replacement therapies, including valve-in-valve procedures, as indicated towards clinical improvement of critically ill patients are possible during the same hospitalization with fewer periprocedural complications. Patients with early failing mitral bioprosthesis pose a therapeutic challenge especially when their clinical condition is critical. VA-ECMO serves as a bridge allowing complicated procedures. With a growing body of evidence in favor of TMViVR especially using the transseptal-transcatheter approach, it is a useful alternate to redo surgery even for critical and high-risk surgical patients. 
